# Sociodemographic Profile and Clinical Outcomes of Gestational Diabetes Mellitus: A Regional Hospital-Based Cross-Sectional Study From Mauritius

**DOI:** 10.7759/cureus.92270

**Published:** 2025-09-14

**Authors:** Sheena Bharati Auckloo, Indrajit Banerjee

**Affiliations:** 1 Department of Internal Medicine, Sir Seewoosagur Ramgoolam Medical College, Vacoas-Phoenix, MUS; 2 Department of Pharmacology, Sir Seewoosagur Ramgoolam Medical College, Vacoas-Phoenix, MUS

**Keywords:** complications in pregnancy, diabetes gestational, diabetes in pregnancy, gestational diabetes insipidus, indian ocean islands, mauritius, pregnancy-related complications

## Abstract

Introduction: Mauritius is among the countries with the highest disease burden globally in diabetes mellitus (DM), ranking twelfth in terms of disease impact. Though data is available for DM in Mauritius, there is a lack of data and research on gestational DM (GDM) in Mauritius. This study aimed to bridge this gap to determine the sociodemographic details of GDM patients in the Mauritian context, the prevalence of GDM in Mauritius, identify the risk factors predisposing Mauritian women to GDM, and assess maternal and fetal/neonatal complications of GDM in Mauritius.

Methods: A cross-sectional study was conducted on pregnant women diagnosed with GDM at Dr. A. G. Jeetoo Hospital, Port Louis, Mauritius, from September 2019 to February 2020.

Results: The mean age of GDM patients was 27.90 ± SD 5.998 years. Most of the patients belonged to the age group 20-30 years (97, 52.2%). Passive smokers accounted for 50 (26.9%). Most of the patients were housewives (97, 52.2%), and the common treatment offered to GDM patients was dietary modification (160, 86%), followed by insulin therapy (16, 8.6%), and metformin in 1 (0.5%). Maternal complications were found in 75 (40.3%) of the patients with GDM. Preterm delivery was the most common type of maternal complication, followed by recurrent urinary tract infection and gestational hypertension. Neonatal complications were present in 60 (32.3%) of the patients. The most common neonatal complication was low birth weight (LBW), followed by hypoglycemia and jaundice.

Conclusion: The prevalence of GDM was found to be 16% in Mauritius. A total of 52.2% patients belonged to the age group of 20-30 years and were housewives (52.2%) by occupation. Most of them followed Christianity (58.6%). About 64.5% of the patients had a positive family history of DM, followed by other comorbidities (10.8%). GDM may cause significant maternal and neonatal complications. This study bridges the existing knowledge gap by determining the prevalence of GDM in Mauritius and identifying the risk factors specific to Mauritian women. The most common neonatal complications were LBW, followed by hypoglycemia and jaundice. The most common maternal complication was preterm delivery followed by recurrent UTI and gestational hypertension.

## Introduction

Gestational diabetes mellitus (GDM) is defined as hyperglycemia that manifests for the first time during pregnancy, which usually resolves shortly after delivery. Early GDM is diagnosed before 20 weeks of gestation, whereas classic GDM typically occurs between 24 and 28 weeks of gestation [1]. According to a recent meta-analysis conducted by Saeedi et al., it was reported that the global prevalence of GDM was 14.7% based on the International Association of Diabetes and Pregnancy Study Groups (IADPSG) criteria [2]. Although the global prevalence of GDM is relatively low, it has been increasing in recent years due to the rising rates of type 2 DM (T2DM), obesity in women, and the advancing maternal age [3]. The prevalence of GDM in a population tends to increase in a direct correlation with the prevalence of T2DM [4]. The prevalence of GDM varies both within and across countries globally, depending on the racial and ethnic composition of the population [5]. According to Kim et al., the overall prevalence of GDM in Florida, United States, was 4.7%, and a significant race predilection has been shown with an increased prevalence in Asian and Pacific Islanders (9.9%), American Indian (6.5%), Hispanic (5.1%), and Non-Hispanic White (4.7% populations compared to Non-Hispanic black populations (4%) [5]. Various other studies point out the ethnic differences in the prevalence of GDM [6].

During early gestation, high insulin sensitivity promotes uptake of glucose by adipocytes in preparation for energy demands in the future. However, as pregnancy progresses, the production of placental hormones such as growth hormone and placental human chorionic somatomammotropin (HCS), as well as local hormones, including estrogen, progesterone, cortisol, and leptin, promotes insulin resistance. This physiological state causes blood glucose to be slightly elevated and to be readily transported to support the growth of the fetus. When the pancreas fails to keep up with the stress of insulin resistance, GDM develops [4,7]. 

The optimal diagnostic criteria for GDM have always been a debate among clinicians around the world. The first diagnostic criteria were proposed by Ryan et al. in the 1960s based on a three-hour 100 g oral glucose tolerance test (OGTT) [8,9]. This was followed by the two-hour 75 g OGTT criteria by the WHO [6]. In 2008, the IADPSG reviewed studies on maternal and fetal outcomes of GDM, including the Hyperglycemia and Adverse Pregnancy Outcomes (HAPO), and subsequently recommended another diagnostic criterion for GDM [10]. The diagnostic criteria for GDM used in Mauritius are the IADPSG. The IADPSG recommends a one-step 75 g OGTT to be performed between 24 and 28 weeks of gestation following an overnight fast. GDM is diagnosed if any one of the following plasma glucose values is met or exceeded: fasting glucose ≥ 92 mg/dL (5.1 mmol/L), one-hour glucose ≥ 180 mg/dL (10.0 mmol/L), or two-hour glucose ≥ 153 mg/dL (8.5 mmol/L). Notably, only one abnormal value is required for diagnosis, making this approach more sensitive compared to previous criteria.

GDM is associated with maternal and fetal complications [11]. Pregnant women with GDM carry a higher risk for hypertensive disorders during pregnancy, including gestational hypertension and preeclampsia. Polyhydramnios is another consequence of GDM, which can result in maternal dyspnea, abnormal fetal presentation, cord prolapse, preterm labor, premature rupture of membranes, and postpartum hemorrhage [12]. Neonates who are born to mothers with GDM are more likely to end up in the neonatal intensive care unit (NICU) as compared to those born to mothers without GDM [13]. GDM can cause macrosomia, which can lead to potential birth injuries, shoulder dystocia, brachial plexus damage, including Erb’s palsy, and fractures of the clavicle and humerus [14].

Mauritius is a tropical island located in the Indian Ocean, with a multiethnic population of 1.2 million. According to the International Diabetes Federation, Mauritius is ranked as the twelfth highest disease-burdened country in the world. Although data is available for DM in Mauritius, there is a lack of data [15]. According to the Noncommunicable Disease Survey Report 2021, the prevalence of T2DM accounts for 19.9% in Mauritius, and it affects one in every five Mauritians [16]. The prevalence is higher in males (21.6%) as compared to females (18.5%) in Mauritius [16]. According to the Health Statistics Report 2023 of Mauritius, DM (23.3%) was the second leading cause of mortality after cardiovascular diseases, with 33%, respectively [17].

GDM in Mauritius has been an unexplored field of study. There is a significant dearth of data on the prevalence, as well as fetal, neonatal, and maternal outcomes related to GDM in Mauritius. The aim of this study is to bridge this knowledge gap to determine the sociodemographic details of GDM patients in the Mauritian context, the prevalence of GDM in Mauritius, identify the risk factors predisposing Mauritian women to GDM, and assess maternal and fetal/neonatal complications of GDM in Mauritius. To the best of our knowledge, this will be the first study conducted to explore the GDM and its associated clinical outcomes in Mauritius.

## Materials and methods

Study design

A cross-sectional study was conducted on pregnant women diagnosed with GDM in Mauritius.

Study setting and data collection 

**Figure 1:**
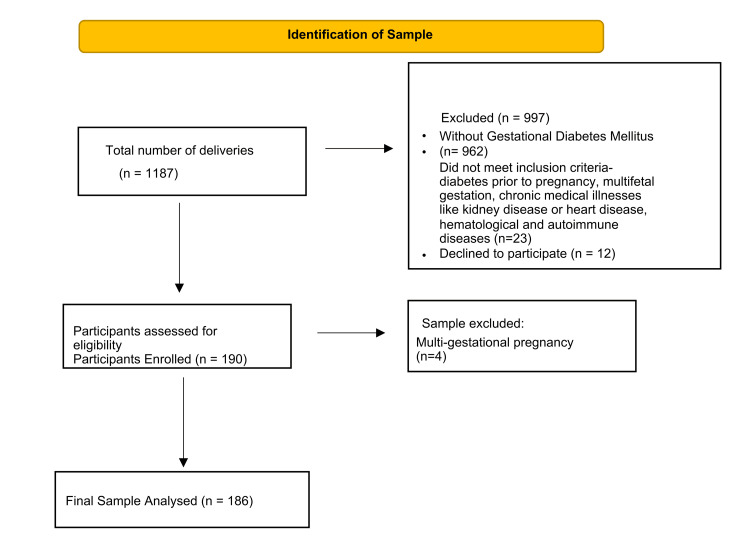
Selection of participants

The study was conducted in the postnatal ward and intensive care unit (ICU) of the Dr. A. G. Jeetoo Hospital, Port Louis, Mauritius, owing to the fact that all mothers postdelivery were admitted to either of these wards. Since all newborns born (n = 190) from GDM mothers are admitted to the nursery or the NICU (Figure 1).

The data was collected from September 2019 to February 2020 at Dr. A. G. Jeetoo Hospital, Port Louis, Mauritius. The data was collected in person by the principal investigator (SBA) by using a prevalidated standardized questionnaire. The investigator received training on administering the questionnaire to ensure consistency across participants. All responses were recorded directly onto the questionnaire forms in a uniform manner to minimize variability and enhance reproducibility. 

Dr. A. G. Jeetoo Hospital, Port Louis, was selected as the place of study, as it is one of the five regional hospitals in Mauritius that serves a large urban and semi-urban population in the country. Given its wide catchment area, it is a key referral center for patients with DM from Port Louis and surrounding areas [18]. The regional hospital, Dr. A. G. Jeetoo Hospital, is situated in the district of Port Louis, which is the capital of Mauritius, and covers area No. 1. It consists of two towns, namely, Port Louis and Beau Bassin, and the following villages: Quartier Militaire, Dagotiere, Camp Thorel, Saint Pierre, Le Hochet, Petite Riviere, Albion, Bambous, St. Julien d’Hotman, L’Esperance, etc. There are also 25 community health centers, six area health centers, and one medi-clinic under the care of Dr. A. G. Jeetoo Hospital.

Inclusion criteria

All the pregnant women with a single-fetus pregnancy, diagnosed with GDM, who delivered at Dr. A. G. Jeetoo Hospital, Mauritius, and those patients who had given informed consent were included in the study. Only single-fetus pregnant women were included in the study due to complications attributed to multifetal gestations.

Exclusion criteria

Patients who had diabetes prior to pregnancy, multifetal gestation, chronic medical illnesses like kidney disease or heart disease, and hematological and autoimmune diseases were excluded from the study. These were excluded as they could be potential confounders of maternal and fetal complications. Patients who declined to provide informed consent were also excluded from the study.

Outcome variables

The main outcome variables of the study were maternal complications (yes/no), type of maternal complications, neonatal complications (yes/no), type of neonatal complications, birth weight (normal weight, underweight, overweight), treatment (diet, insulin, metformin, no treatment), and type of delivery (assisted delivery, elective cesarian section, emergency cesarian section, normal vaginal delivery).

Explanatory variables

Variables assessed at individual levels were age (<20 years, 20-30 years, >30 years), education level (no education, primary, secondary, tertiary), occupation (housewife, professional, skilled worker, unskilled worker), ethnic background (Christian, Hindu, Muslim), marital status (cohabitating, married, single), family income (<9000 MUR/month, 9000-25000 MUR/month, >25000 MUR/month), smoking (ex-smoker, nonsmoker, passive, smoker), alcohol (no/yes), parity (multiparous, uniparous, or primigravida), past GDM (yes/no), and obesity (normal weight, obese class 1, obese class 2, obese class 3, overweight, underweight).

Questionnaire design

The questionnaire was designed after a thorough literature review. The questionnaire was divided into three sections: (A) sociodemographic details of patients with GDM, (B) risk factors of GDM, and (C) clinical outcome of patients with GDM (Appendix). 

Questionnaire validity and reliability

The questionnaire was validated by four experts in the fields of diabetes, endocrinology, public health, and obstetrics and gynecology. The average congruency percentage was found to be 90% according to the methodology laid down by Polit and Beck [19]. The questionnaire was refined based on their feedback. Prior to administering the final questionnaire, a pilot study was conducted among 10 patients for the readability and face validity. For reliability analysis, alpha Cronbach’s was applied, and it was found to be 0.822. 

Sample size calculation

The number of sample sizes required to attain results that could be applied to the general population. It was statistically determined by using the formula:

 $n = z^2 p (1-p)/d^2$

where n = required sample size, z = desired confidence interval of 1.96 at 95% confidence interval, p = expected prevalence- 0.14 or 14%, and d = absolute precision, which is set at 0.05 or 5%:

$n = 1.96 ^2 X 0.14(1-0.14)/0.05^2$

The sample size was found to be 185 patients with GDM. A total of 186 participants were enrolled, meeting the required sample size.

Minimizing the bias

To minimize potential sources of bias, data were collected using a prevalidated questionnaire to ensure and reduce measurement bias. Selection bias was addressed through appropriate sampling techniques aimed at achieving a representative population. Confounding was controlled during statistical analysis by adjusting for key variables, including age, education level, occupation, ethnic background, marital status, family income, smoking, alcohol use, parity, history of GDM, and BMI. These steps were taken to enhance the validity and generalizability of the study findings.

Data management and statistical analysis

MS Excel (Microsoft Corporation, Redmond, Washington, United States) and IBM SPSS Statistics for Windows, Version 28 (Released 2021; IBM Corp., Armonk, New York, United States) were used to analyze the data. Descriptive statistical analysis was used. The Chi-square test was used to find associations between the explanatory and outcome variables. A p-value of <0.05 was considered to be statistically significant. 

Ethics clearance

Prior to the study, an application for ethical clearance to the National Ethics Committee of the Ministry of Health and Quality of Life (MOHQL) was made. Once approved, data collection was carried out. Approval was obtained on November 27, 2018 (Reference no: MHC/CT/NETH/AUCKL) from the Ethics Subcommittee.

## Results

Out of a total of 1187 deliveries, 16% (n = 190) were identified as GDM patients. Among the 190 GDM patients, four were excluded as they had a multigestational pregnancy. Therefore, 186 GDM patients were included in the study.

Sociodemographic details

The mean age of the GDM patients was 27.90 ± SD 5.998 years. Most of the patients belonged to the age group 20-30 years (97, 52.2%) and studied up to secondary level (149, 80.1%). While most of the patients were housewives (97, 52.2%), the second most common job was unskilled workers (48, 25.8%). As far as religion is concerned, most of them followed Christianity 109 (58.6%). A total of 144 (77.4%) were married, while 39 (21%) had a live-in relationship. Passive smokers accounted for 50 (26.9%), smokers for 25 (13.4%), ex-smokers for six (3.2%), and nonsmokers for 105 (56.5%). About 120 (64.5%) of the patients had a positive family history of DM, followed by other comorbidities in 20 (10.8%). A total of 26 patients (14%) had a past history of GDM. Concerning the weight of the GDM patients, 56 (30.1%) were overweight, 44 (23.7%) were obese class 1, 12 (6.5%) were obese class 2, 10 (5.4%) were obese class 3, eight (4.3%) were underweight, and 56 (30.1%) had a normal body weight (Table 1).

**Table 1:** Sociodemographic details of patients with gestational diabetes mellitus in Mauritius, September 2019 to February 2020 (N = 186 cases) MUR: Mauritian Rupees

Age	Frequency n (%)	95% confidence interval (%)
<20 years	25 (13.4)	(8.8, 18.0)
20-30 years	97 (52.2)	(45.0, 59.3)
>30 years	64 (34.4)	(27.5, 41.3)
Education level		
No education	2 (1.1)	(0, 2.7)
Primary	25 (13.4)	(8.8, 18.0)
Secondary	149 (80.1)	(74.3, 85.8)
Tertiary	10 (5.4)	(2.2, 8.6)
Occupation		
Housewife	97 (52.2)	(45.0, 59.3)
Professional	23 (12.4)	(7.6, 17.1)
Skilled worker	18 (9.7)	(5.4, 14.0)
Unskilled worker	48 (25.8)	(19.5, 32.1)
Ethnic background		
Christian	109 (58.6)	(51.6, 65.6)
Hindu	36 (19.4)	(13.4, 25.4)
Muslim	41 (22)	(16.0, 28.0)
Marital status		
Cohabitating	39 (21)	(15.0, 27.0)
Married	144 (77.4)	(71.5, 83.3)
Single	3 (1.6)	(0, 3.4)
Family income		
<9000 MUR/month	94 (50.5)	(43.4, 57.6)
9000-25000 MUR/month	65 (34.9)	(28.1, 41.7)
>25000 MUR/month	27 (14.5)	(9.4, 19.6)
Family history		
Diabetes mellitus	120 (64.5)	(57.7, 71.3)
Other comorbidities	20 (10.8)	(6.3, 15.3)
Parity		
Multiparous	67 (36)	(26.6, 40.0)
Nulliparous	62 (33.3)	(29.2, 42.8)
Uniparous	57 (30.6)	(24.0, 37.2)
Past gestational diabetes mellitus		
No	160 (86)	(81.1, 90.9)
Yes	26 (14)	(9.1, 18.9)
Obesity		
Normal weight	56 (30.1)	(23.6, 36.6)
Obese class 1	44 (23.7)	(17.5, 29.9)
Obese class 2	12 (6.5)	(2.9, 10.1)
Obese class 3	10 (5.4)	(2.2, 8.6)
Overweight	56 (30.1)	(23.6, 36.6)
Underweight	8 (4.3)	(1.4, 7.2)
ICU		
No	182 (97.8)	(95.9, 99.7)
Yes	4 (2.2)	(0.3, 4.1)
Smoking		
Ex smoker	6 (3.2)	(0.7, 5.7)
Non smoker	105 (56.5)	(49.3, 63.6)
Passive	50 (26.9)	(20.4, 33.4)
Smoker	25 (13.4)	(8.8, 18.0)
Alcohol		
No	175 (94.1)	(90.5, 97.7)
Yes	11 (5.9)	(2.3, 9.5)

Sociodemographic profile of patients with GDM and clinical outcomes

Maternal complications were found in 75 (40.3%) of the patients with GDM. Neonatal complications were present in 60 (32.3%) of the patients. The mean birth weight of the neonate was found to be 2.832 ± SD A0.531 kg. A total of 39 (21%) of the neonates were underweight, four (2.2%) were overweight, whereas 143 (76.9%) were of normal weight. Table 2 signifies the cross-tabulation between various sociodemographic profiles of patients with maternal and neonatal complications and the birth weight of neonates. 

**Table 2:** Sociodemographic details of patients with gestational diabetes mellitus and maternal complications, neonatal complications, and birth weight in Mauritius, September 2019 to February 2020 (N = 186 cases) χ²: Chi-square; x: p >0.05 (statistically insignificant) Chi-square test used to calculate p-values

Sociodemographic details	Maternal complications n (%)		χ² value	p-value	Neonatal complications n (%)		χ² value	p-value	Birth weight n (%)			χ² value	p-value
Age	No 111 (59.7)	Yes 75 (40.3)	11.037	0.2^x^	No 126 (67)	Yes 60 (32.3)	2.097	0.35^x^	Normal weight 143 (76.9)	Overweight 4 (2.2)	Underweight 39 (21)	6.375	0.173^x^
<20 years	19 (17.1)	6 (8)			14 (11.1)	11 (18.3)			17 (11.9)	0 (0)	8 (20.5)		
20-30 years	55 (49.5)	27 (36)			43 (34.1)	21 (35)			81 (56.6)	2 (50)	17 (43.6)		
>30 years	37 (33.3)	42 (56)			69 (54.8)	28 (46.7)			45 (31.50	2 (50)	14 (35.9)		
Education level													
No education	2 (1.8)	0 (0)	12.278	0.187^x^	2 (1.6)	0 (0)	1.422	0.7^X^	2 (1.4)	0 (0)	0 (0)	4.199	0.65^x^
Primary	19 (17.1)	6 (8)			16 (12.7)	9 (15)			19 (13.3)	0 (0)	6 (15.4)		
Secondary	84 (75.7)	65 (86.7)			102 (81)	47 (78.3)			115 (80.4)	3 (75)	31 (79.5)		
Tertiary	6 (5.4)	4 (5.3)			6 (4.8)	4 (6.7)			7 (4.9)	1 (25)	2 (5.1)		
Occupation													
Housewife	54 (48.6)	43 (57.3)	8.656	0.363^X^	64 (50.8)	33 (55)	2.660	0.447^x^	74 (51.7)	1 (25)	22 (56.4)	5.534	0.477^x^
Professional	12 (10.8)	11 (14.7)			14 (11.1)	9 (15)			15 (10.5)	1 (25)	7 (17.9)		
Skilled worker	12 (10.8)	6 (8)			15 (11.9)	3 (5)			16 (11.2)	0 (0)	2 (5.1)		
Unskilled worker	33 (29.7)	15 (20)			33 (26.2)	15 (25)			38 (26.6)	2 (50)	8 (20.5)		
Ethnic background													
Christian	70 (63.1)	39 (52)	6.658	0.3^x^	73 (57.9)	36 (60)	0.827		83 (58)	4 (100)	22 (56.4)	3.272	0.513^x^
Hindu	20 (18)	16 (21.3)			23 (18.3)	13 (21.7)		0.661^x^	27 (18.9)	0 (0)	9 (23.1)		
Muslim	21 (18.9)	20 (26.7)			30 (23.8)	11 (18.3)			33 (23.1)	0 (0)	8 (20.5)		
Marital status													
Cohabitating	23 (20.7)	16 (21)	2.182	0.357^X^	24 (19)	15 (25)	2.691	0.26^X^	30 (21)	2 (50)	7 (17.9)	2.512	0.642^x^
Married	85 (76.6)	59 (78.7)			101 (80.2)	43 (71.7)			111 (77.6)	2 (50)	31 (79.5)		
Single	3 (2.7)	0(0)			1 (0.8)	2 (3.3)			2 (1.4)	0(0)	1 (2.6)		
Family income													
<9000 MUR/month	60 (54.1)	34 (45.3)	10.591	0.451^x^	58 (46)	36 (60)	3.292	0.183^X^	69 (48.3)	1 (25)	24 (61.5)	4.105	0.392^x^
9000-25000 MUR/month	35 (31.5)	30 (40)			49 (38.9)	16 (26.7)			54 (37.8)	2 (50)	9 (23.1)		
>25000 MUR/month	16 (14.4)	11 (14.7)			19 (15.1)	8 (13.3)			20 (14)	1(25)	6 (15.4)		

The treatment offered most commonly was dietary modification (160, 86%), followed by insulin therapy (16, 8.6%), and metformin in one (0.5%). Nine (4.8%) patients did not follow any treatment. Normal vaginal delivery was the most common mode of delivery (70, 27.6%), followed by elective cesarean section (52, 28%), emergency cesarean section (52, 28%), and assisted delivery (2, 1.1%).

Dietary modification was the preferred treatment across all age groups (160, 86%). Metformin was used in only one patient belonging to the age group 20-30 years. It should be noted that insulin treatment was mostly used in the age group >30 years (12, 75%). The group of patients who did not follow any treatment belonged to the age group <20 years (4, 44.4%). It was found to be statistically significant (p < 0.05).

There was an association between the type of delivery and the age group. In the age groups <20 years and 20-30 years, normal vaginal delivery was the most common mode of delivery with 13 (18.6%) and 40 (57.1%), respectively. In the age group >30 years, elective cesarean section was the most preferred method of delivery (29, 46.8%). This finding was found to be statistically significant (p < 0.05) (Table 3). 

**Table 3:** Cross-tabulation between sociodemographic details of patients with gestational diabetes mellitus and treatment, type of delivery in Mauritius, September 2019 to February 2020 (N = 186 cases) CS: cesarean section; χ²: Chi-square; x: p > 0.05 (statistically insignificant); **: p < 0.05 (statistically significant) Chi-square test used to calculate p-values

Sociodemographic details	Treatment n (%)				χ² value	p-value	Type of delivery n (%)				χ² value	p-value
Age	Diet 160 (86)	Insulin 16 (8.6)	Metformin 1 (0.5)	No treatment 9 (4.8)	21.273	0.002**	Assisted delivery 2 (1.1)	Elective CS 62 (33.3)	Emergency CS 52 (28)	Normal vaginal delivery 70 (37.6)	22.192	0.001**
<20 years	21 (13.1)	0 (0)	0 (0)	4 (44.4)			2 (100)	4 (6.5)	4 (6.5)	13 (18.6)		
20-30 years	89 (55.6)	4 (25)	1 (100)	3 (33.3)			0 (0)	29 (46.8)	28 (53.8)	40 (57.1)		
>30 years	50 (31.3)	12 (75)	0 (0)	2 (22.2)			0 (0)	29 (46.8)	18 (34.6)	17 (24.3)		
Education level												
No education	2 (1.3)	0 (0)	0 (0)	0 (0)	3.776	0.926^X^	0 (0)	1 (1.6)	0 (0)	1 (1.4)	5.346	0.803^X^
Primary	19 (11.9)	4 (25)	0 (0)	2 (22.2)			0 (0)	7 (11.3)	5 (9.6)	13 (18.6)		
Secondary	130 (81.3)	11 (68.8)	1 (100)	7 (77.8)			2 (100)	49 (79)	44 (84.6)	54 (77.1)		
Tertiary	9 (5.6)	1 (6.3)	0 (0)	0 (0)			0 (0)	5 (8.1)	3 (5.8)	2 (2.9)		
Occupation												
Housewife	78 (48.8)	11 (68.8)	0 (0)	8 (88.9)	15.997	0.067^x^	2 (100)	26 (41.9)	25 (48.1)	44 (62.9)	8.846	0.452^x^
Professional	22 (13.8)	0 (0)	1 (100)	0 (0)			0 (0)	11 (17.7)	6 (11.5)	6 (8.6)		
Skilled worker	17 (10.6)	1 (6.3)	0 (0)	0 (0)			0 (0)	7 (11.3)	6 (11.5)	5 (7.1)		
Unskilled worker	43 (26.9)	4 (25)	0 (0)	1 (11.1)			0 (0)	18 (29)	15 (28.8)	15 (21.4)		
Ethnic background												
Christian	92 (57.5)	11 (68.8)	0 (0)	6 (66.7)	7.213	0.302^x^	1 (50)	11 (17.7)	14 (26.9)	13 (18.6)	2.596	0.657^X^
Hindu	30 (18.8)	4 (25)	1 (100)	1 (11.1)			1 (50)	50 (80.6)	38 (73.1)	55 (78.6)		
Muslim	38 (23.8)	1 (6.3)	0 (0)	2 (22.2)			0 (0)	1 (1.6)	0 (0)	2 (2.9)		
Marital status												
Cohabitating	36 (22.5)	2 (12.5)	0 (0)	1 (11.1)	2.336	0.886^X^	1 (50)	11 (17.7)	14 (26.9)	13 (18.6)	4.142	0.657^X^
Married	121 (75.6)	14 (87.5)	1 (100)	8 (88.9)			1 (50)	50 (80.6)	38 (73.1)	55 (78.6)		
Single	3 (1.9)	0 (0)	0 (0)	0 (0)			0(0)	1 (1.6)	0 (0)	2 (2.9)		
Family income												
<9000 MUR/month	79 (49.4)	9 (56.3)	0 (0)	6 (66.7)	8.730	0.189^x^	2 (100)	30 (48.4)	23 (44.2)	39 (55.7)	9.502	0.858^x^
9000-25000 MUR/month	56 (35)	6 (37.5)	0 (0)	3 (33.3)			0 (0)	20 (32.3)	18 (34.6)	27 (38.6)		
>25000 MUR/month	25 (15.6)	1 (6.3)	1 (100)	0 (0)			0 (0)	12 (19.4)	11 (21.2)	4 (5.7)		

Risk factors and clinical outcomes

Among the patients who developed maternal complications, most of the patients were nonsmokers (60, 54.1%), followed by passive smokers, which accounted for 19 (25.3%). Most of those who had maternal complications were overweight (24, 32%), followed by normal weight (21, 28%) and obese class 1 (16, 21.3%). Neonatal complications were most common in overweight mothers (21, 35%). Neonatal complications were found in multiparous women (29, 48.3%), followed by nulliparous women (21, 35%) and uniparous women (10, 16.7%). This was found to be statistically significant (p < 0.05) (Table 4). 

**Table 4:** Correlation between risk factors of patients with gestational diabetes mellitus and maternal complications, neonatal complications, and birth weight in Mauritius, September 2019 to February 2020 (N = 186 cases) χ²: Chi-square; x: p > 0.05 (statistically insignificant); **: p < 0.05 (statistically significant) Chi-square test used to calculate p-values

Risk factors	Maternal complications n (%)		χ² value	p-value	Neonatal complications n (%)		χ² value	p-value	Birth weight n (%)			χ² value	p-value
Smoking	No 111 (59.7)	Yes 75 (40.3)	0.708	0.871^x^	No 126 (67)	Yes 60 (32.3)	4.469	0.215^X^	Normal weight 143 (76.9)	Overweight 4 (2.2)	Underweight 39 (21)	5.419	0.491^x^
Ex smoker	4 (3.6)	2 (2.7)			3 (2.4)	3 (5)			4 (2.8)	0 (0)	2 (5.1)		
Nonsmoker	60 (54.1)	45 (60)			75 (59.5)	30 (50)			83 (58)	3 (75)	19 (48.7)		
Passive	31 (27.9)	19 (25.3)			35 (27.8)	15 (25)			40 (28)	1 (25)	9 (23.1)		
Smoker	16 (14.4)	9 (12)			13 (10.3)	12 (20)			16 (11.2)	0 (0)	9 (23.1)		
Alcohol													
No	103 (92.8)	72 (96)	0.827	0.53^x^	121 (96)	54 (90)	2.658	0.103^x^	136 (95.1)	4 (100)	35 (89.7)	1.840	0.399^x^
Yes	8 (7.2)	3 (4)			5 (4)	6 (10)			7 (4.9)	0 (0)	4 (10.3)		
Parity													
Nulliparous	34 (30.6)	28 (37.3)	1.252	0.535^X^	41 (32.5)	21 (35)	9.448	0.009**	46 (32.2)	2 (50)	14 (35.9)	3.092	0.543^x^
Multiparous	40 (36)	27 (36)			38 (30.2)	29 (48.3)			49 (34.3)	1 (25)	17 (43.6)		
Uniparous	37 (33.3)	20 (26.7)			47 (37.3)	10 (16.7)			48 (33.6)	1 (25)	8 (20.5)		
Past gestational diabetes mellitus													
No	95 (85.6)	65 (86.7)	0.044	0.835^X^	107 (84.9)	53 (88.3)	0.394	0.530x	124 (86.7)	4 (100)	32 (82.1)	1.218	0.544^x^
Yes	16 (14.4)	10 (13.3)			19 (15.1)	7 (11.7)			19 (13.3)	0(0)	7 (17.9)		
Obesity													
Normal weight	35 (31.5)	21(28)	3.478	0.627^x^	39 (31)	17 (28.3)	1.358	0.929X	42 (29.4)	0 (0)	14 (35.9)	10.188	0.424^x^
Obese class 1	28 (25.2)	16 (21.3)			31 (24.6)	13 (21.7)			37 (25.9)	1 (25)	6 (15.4)		
Obese class 2	6 (5.4)	6 (8)			9 (7.1)	3 (5)			9 (6.3)	0 (0)	3 (7.7)		
Obese class 3	4 (3.6)	6 (8)			7 (5.6)	3 (5)			10 (7)	0 (0)	0 (0)		
Overweight	32 (28.8)	24 (32)			35 (27.8)	21 (35)			39 (27.3)	3 (75)	14 (35.9)		
Underweight	6 (5.4)	2 (2.7)			5 (4)	3 (5)			6 (4.2)	0 (0)	2 (5.1)		

There was an association between the type of treatment provided and the past history of GDM. In GDM patients with no past history of GDM in earlier pregnancies, 141 (88.1%) were treated by diet, 10 (62.5%) received insulin therapy, and one (100%) received metformin with no past history of GDM (p < 0.05) (Table 5).

**Table 5:** Correlation between risk factors of patients with gestational diabetes mellitus and treatment, type of delivery in Mauritius, September 2019 to February 2020 (N = 186 cases) CS: cesarean section; χ²: Chi-square; x: p >0.05 (statistically insignificant); **: p < 0.05 (statistically significant) Chi square test used to calculate p-values

Risk factors	Treatment n (%)				χ² value	p-value	Type of delivery n (%)				χ² value	p-value
Smoking	Diet 160 (86)	Insulin 16 (8.6)	Metformin 1 (0.5)	No treatment 9(4.8)	8.995	0.438^X^	Assisted delivery 2 (1.1)	Elective CS 62 (33.3)	Emergency CS 52 (28)	Normal vaginal delivery 70 (37.6)	4.890	0.844^X^
Ex smoker	6 (3.8)	0 (0)	0 (0)	0(0)			0 (0)	3 (4.8)	2 (3.8)	1 (1.4)		
Non smoker	90 (56.3)	11 (68.8)	0 (0)	4 (44.4)			1 (50)	32 (51.6)	34 (65.4)	38 (54.3)		
Passive	45 (28.1)	2 (12.5)	1 (100)	2 (22.2)			1 (50)	17 (27.4)	11 (21.2)	21 (30)		
Smoker	19 (11.9)	3 (18.8)	0(0)	3 (33.3)			0 (0)	10 (16.1)	5 (9.6)	10 (14.3)		
Alcohol												
Yes	8 (5)	1(6.3)	0 (0)	2 (22.2)	4.608	0.203^x^	0 (0)	1 (1.6)	3 (5.8)	7 (10)	4.289	0.232^x^
No	152 (95)	15 (93.8)	1 (100)	7 (77.8)			2 (100)	61 (98.4)	49 (94.2)	63 (90)		
Parity												
Nulliparous	51 (31.9)	8 (50)	1(100)	2 (22.2)	4.888	0.558^X^	0 (0)	21 (33.9)	17 (32.7)	24 (34.3)	10.653	0.10^x^
Multiparous	58 (36.3)	5 (31.3)	0 (0)	4 (44.4)			1 (50)	16 (25.8)	26 (50)	24 (34.3)		
Uniparous	51 (31.9)	3 (18.8)	0 (0)	3 (33.3)			1 (50)	25 (40.3)	9 (17.3)	22 (31.4)		
Past gestational diabetes mellitus												
No	141 (88.1)	10 (62.5)	1 (100)	8 (88.9)	8.175	0.043**	2 (100)	50 (80.6)	46 (88.5)	62 (88.6)	2.451	0.484^X^
Yes	19 (11.9)	6 (37.5)	0 (0)	1 (11.1)			0 (0)	12 (19.4)	6 (11.5)	8 (11.4)		
Obesity												
Normal weight	49 (30.6)	1 (6.3)	0 (0)	6 (66.7)	20.906	0.140^X^	1 (50)	13 (21)	12(23.1)	30 (42.9)	19.288	0.140^x^
Obese class 1	35 (21.9)	7 (43.8)	0 (0)	2 (22.2)			0 (0)	21 (33.9)	11 (21.2)	12 (17.1)		
Obese class 2	12 (7.5)	0 (0)	0 (0)	0 (0)			0 (0)	7 (11.3)	4 (7.7)	1 (1.4)		
Obese class 3	8 (5)	2 (12.5)	0 (0)	0 (0)			0 (0)	3 (4.8)	4 (7.7)	3 (4.3)		
Overweight	49 (30.6)	6 (37.5)	1 (100)	0 (0)			1 (50)	16 (25.8)	19 (36.5)	20 (28.6)		
Underweight	7 (4.4)	0 (0)	0 (0)	1 (11.1)			0 (0)	2 (3.2)	2 (3.8)	4 (5.7)		

Type of maternal complications

**Figure 2:**
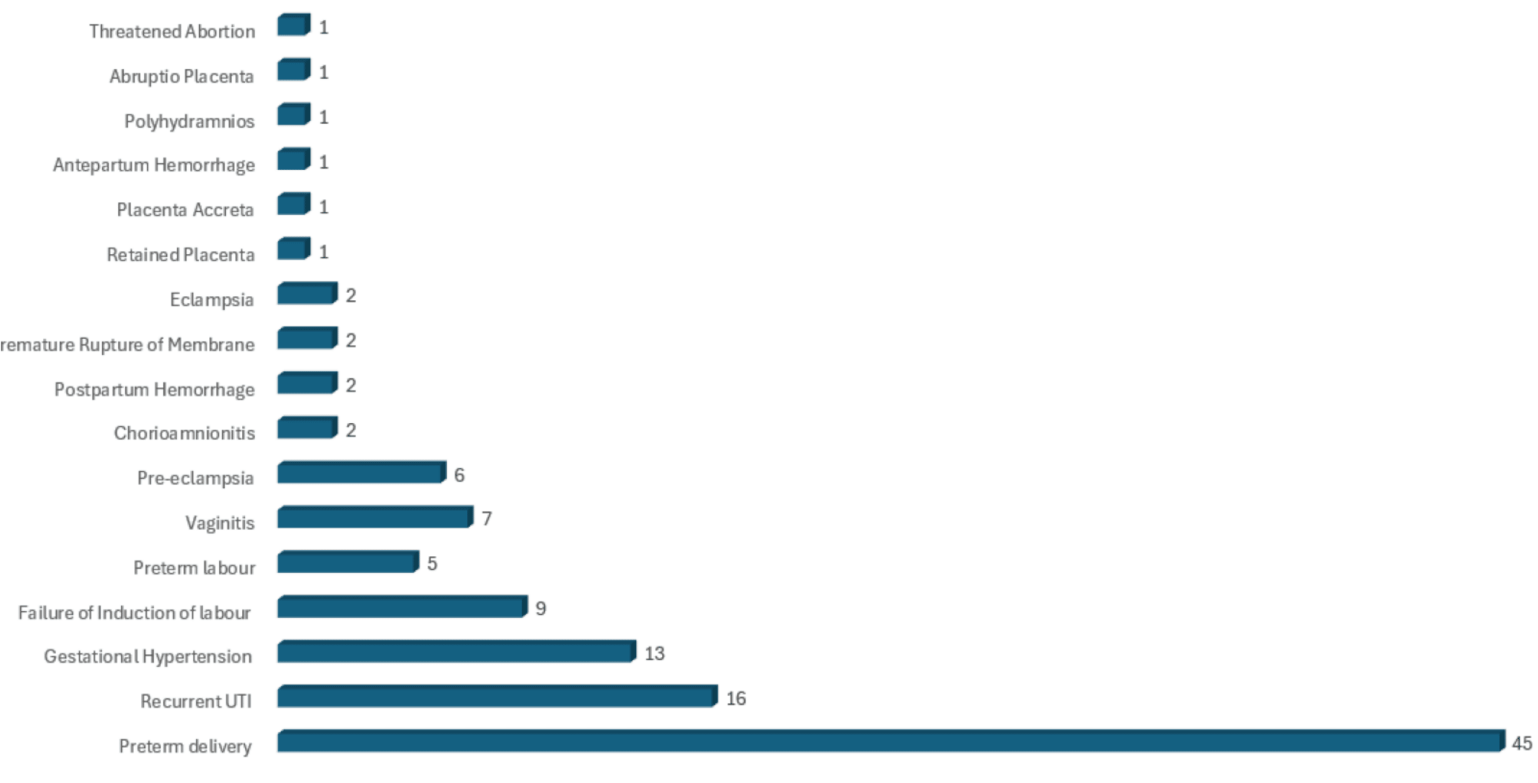
Type of maternal complications with gestational diabetes mellitus in Mauritius, September 2019 to February 2020 (N = 186 cases)

Maternal complications were found in 75 (40.3%) of the patients with GDM. Most patients had more than one complication. Preterm delivery(45) was the most common type of maternal complication, followed by recurrent urinary tract infection (16) and gestational hypertension (13). Other types of complications are depicted in Figure 2.

Type of neonatal complications

**Figure 3:**
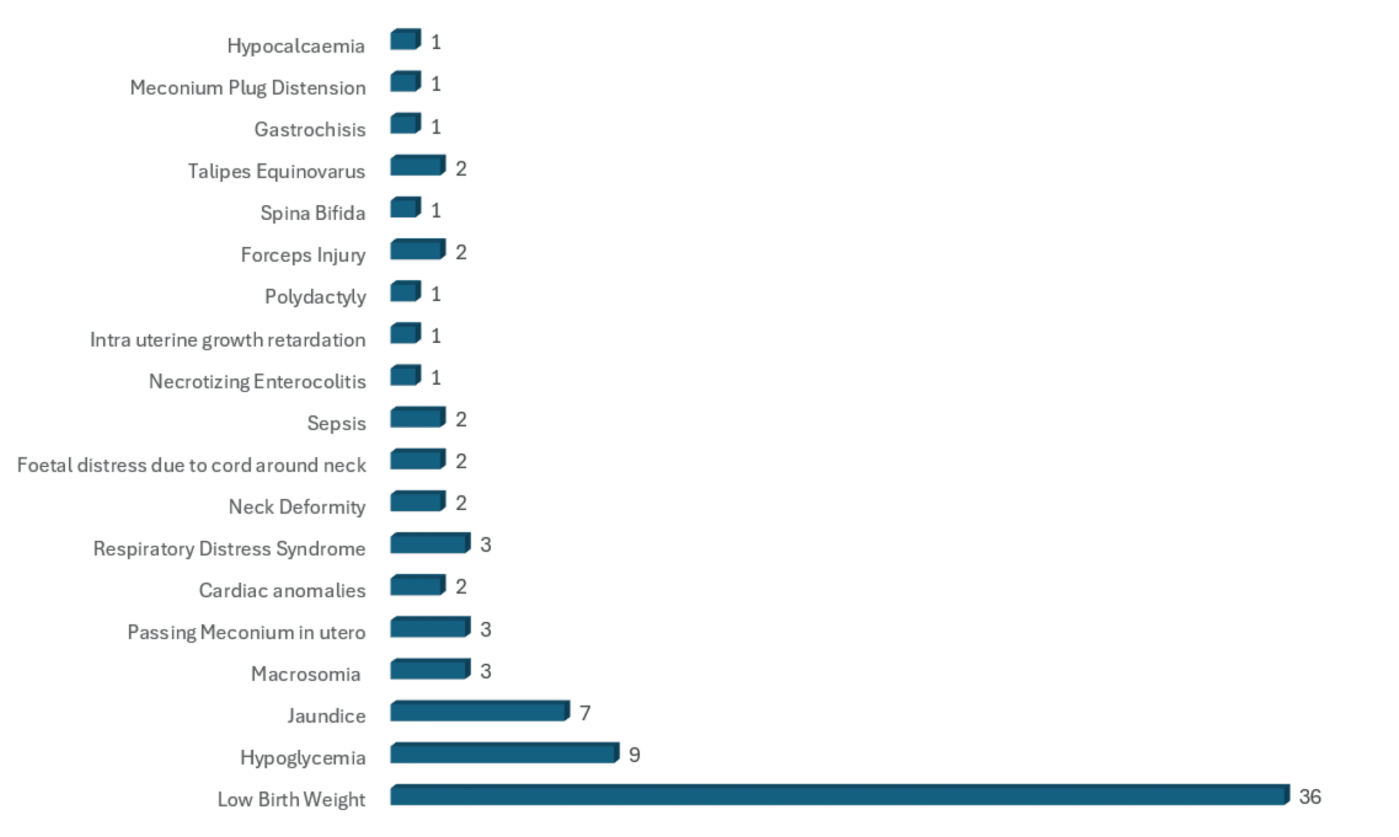
Type of neonatal complications with gestational diabetes mellitus in Mauritius, September 2019 to February 2020 (N = 186 cases)

Neonatal complications were present in 60 (32.3%) of the patients. Most patients had more than one complication. The most common neonatal complication was low birth weight (LBW), which was reported in 36 patients, followed by hypoglycemia (9) and jaundice. Other types of complications are shown in Figure 3.

## Discussion

Prevalence of GDM

During this prospective study over six months, the prevalence of GDM was found to be 16% in Mauritius. This finding was similar to the global prevalence of GDM, which accounted for 14.7% [2]. In India, the prevalence of GDM has increased from 2% in 1980, 7% in 1990, to 16.55% in 2000 [20]. Our results show a relatively higher prevalence of GDM (16%) as compared to the other studies conducted in India. As the prevalence of diabetes in the world is increasing, the prevalence of GDM is also increasing. This could be explained by our genetic predisposition to diabetes and our westernized lifestyle.

Sociodemographic profile

The mean age of the GDM patients was 27.90 ± SD 5.998 years. Most of the patients belonged to the age group 20-30 years (97, 52.2%). Shukla et al. showed that out of 50 GDM patients, 82% (n = 41) were older than 25 years old, while only 18% (n = 9) were below the age of 25 years old. The mean age was 29 years [21]. Kalra et al. showed that compared to women without GDM, women with GDM were significantly older, with the mean ages being 24.7 ± 3.11 years and 27.1 ± 2.44 years, respectively. Thus, our results are in line with the other reports showing that GDM is more prevalent among older women [22]. The majority of GDM women studied up to secondary level (149, 80.1%). Bouthoorn et al. showed that after adjusting for age, family history of diabetes, and parity, women with low educational level were three times more prone to develop GDM than women with the highest level of education. The study further concluded that low education is linked to GDM mainly due to higher rates of overweight and obesity [23]. However, in another meta-analysis by Wang et al., it was concluded that there was no such association between the level of education and GDM. Our results are more in line with the latter, as the majority of the GDM population studied up to the upper secondary level [24]. While most of the patients were housewives (97, 52.2%), the second most common job was as unskilled workers (48, 25.8%). Our findings are similar to a study conducted by Khan et al., which states that GDM is more common in housewives (75.7%) [25]. According to Ali et al., women in the lowest income group had significantly higher rates of GDM as compared to women in the highest family income group. Our results are in line with most of the studies showing that GDM tends to be more prevalent in people with lower socioeconomic status [26]. As far as religion is concerned, most of them followed Christianity (109, 58.6%). According to Sreelakshmi et al., the prevalence of GDM was found to be higher in Blacks, Latinos, Native Americans, and Asian women than in White women [6]. Our results show a higher prevalence of Christians and Muslims in the GDM sample. However, it should be noted that Jeetoo Hospital is situated in the center of the capital, serving multiple suburbs, where Muslims and Christians form the majority of the population. About 120 (64.5%) of the patients had a positive family history of DM, followed by other comorbidities (20, 10.8%). It should be noted that only family history in first-degree relatives was considered in our study. In a study conducted by Dudhwadkar, it was reported that 20% of the GDM had a positive family history of DM in their first-degree relative [27].

Sreelakshmi et al. found that nearly half (48.3%, n = 60) of the participants with GDM had a positive family history for T2DM. Thus, as portrayed by other studies, our results also show that a significant portion of our GDM sample had a family history of DM [6]. Passive smokers accounted for 50 (26.9%). Our findings were similar to a meta-analysis conducted by Zhang et al., which had similar findings and concluded that passive smoking increases the risk of GDM [28]. Concerning the weight of the GDM patients, 56 (30.1%) were overweight. In a study by Martin et al., it was observed that the prevalence of GDM increased with the maternal BMI (6.74%) in overweight, 13.42% in obese class 1, 12.79% in obese class 2, and 20% in obese class 3 [29]. According to a meta-analysis by Lee et al., it was found that the chances of GDM were increased by a history of previous GDM, congenital abnormalities, and macrosomia [30]. In a study by Kalra et al., it was shown that 15.15% of GDM patients had a previous history of fetal or early neonatal deaths [22]. Our results are in line with the previous studies showing that there’s a strong association with previous fetal/neonatal and maternal complications and the prevalence of GDM.

Type of management

The most common treatment offered to GDM patients was dietary modifications (160, 86%), followed by insulin therapy (16, 8.6%) and metformin in one (0.5%), whereas nine (4.8%) patients did not follow any treatment. The GDM treatment is set according to the needs of each patient. It is stated that after a GDM diagnosis, all patients should follow a diet and physical exercise regimen. Approximately 70-80% cases of GDM can be controlled with changes in lifestyle. If the target is not achieved within two weeks, treatment should be shifted to pharmacotherapy. The first line of pharmacological treatment is insulin, followed by metformin and glibenclamide for those who are resistant to insulin [31].

Type of delivery among GDM patients

Normal vaginal delivery (NVD) was the most common mode of delivery with 70 (27.6%) cases, followed by elective cesarian section with 52 (28%), emergency CS with 52 (28%), and assisted delivery with two (1.1%). GDM is not an absolute indication for CS. It is advised to schedule a CS in case of fetal weight >4500g, knowing the increased prevalence of complications if NVD is performed. GDM patients without any previous obstetric complications (perinatal death, macrosomia, PIH) should wait for spontaneous delivery [31]. Dudhwadkar et al. reported that 52% (n = 26) of patients underwent the LSCS (34% (n = 17) elective and 18% (n = 9) emergency), 46% (n = 22) delivered vaginally, and 2% (n = 1) delivered by vacuum-assisted delivery [27].

Our study is in general agreement with studies conducted in third-world countries, showing that the rate of CS as a mode of delivery is quite high among GDM women in Mauritius. In fact, the number of CS in our sample (61.29%) is even higher than all the previous studies (11.7%, 12.6%, 52%, 58%, and 40%). This may be due to a higher rate of risky obstetric histories among our GDM mothers.

Distribution of birth weights among neonates born to GDM mothers

The mean birth weight of the neonate was found to be 2.832 ± SD A0.531 kg. A total of 39 (21%) of the neonates were underweight, four (2.2%) were overweight, whereas 143 (76.9%) were normal-weight neonates. According to Makgoba et al., birth weight is considered an essential result of pregnancy as it reflects the growth profile of the fetus but has an impact on the infant, childhood, and adult morbidity and mortality. Birth weight depends on genetic factors (e.g., race), but it is also affected by constitutional/environmental factors such as maternal age, BMI, DM, smoking, and social status [32].

Maternal complications

In our study (n = 4,5), GDM women had preterm deliveries and were the most common type of maternal complication, followed by recurrent urinary tract infections (16) and gestational hypertension (13). Our findings are similar to a study conducted by Karkia et al., which reported that preterm delivery is a major complication of GDM [12]. Kari et al. state that there is a direct link between DM (including GDM and DM) and premature rupture of the membrane. The most important complication of premature rupture of the membrane (PROM) is chorioamnionitis (intra-amniotic infection), which may result in fetal complications such as abruptio placentae, fetal distress, fetal restrictive deformities, pulmonary hypoplasia, preterm birth, and fetal/newborn death [33].

This relationship was seen in our study, as the (n = 2) PROM reported in our sample were followed by chorioamnionitis. One case of chorioamnionitis was linked to several neonatal complications, including respiratory distress syndrome, sepsis, hypotension, persistent metabolic acidosis, conjunctivitis, hypocalcemia, and a LBW. The second case of chorioamnionitis was linked to neonatal LBW only. Recurrent urinary tract infection (n = 16) was the second most common type of complication found in this study. This finding is parallel to the study conducted by Rizk et al. [34]. Gestational hypertension (n = 13) was the third most common complication. According to Aziz et al., it was found that GDM is associated with gestational hypertension and poses a risk of developing preeclampsia [35]. In our study, two cases of PPH were reported, of which 1 was admitted to the ICU. Both cases in our study were due to uterine atonicity. One case of retained placenta was also recorded, which may be linked to uterine atonicity, as the most common cause of retained placenta is an atonic uterus. Causes of an atonic uterus that could be linked to GDM include grand multipara, overdistension of the uterus (polyhydramnios and macrosomia), as well as obesity (BMI > 35), age (>40), among others. Muche et al. demonstrated in their study that GDM women were five times more at risk of developing PPH compared to non-GDM women. The article related its causes to fetal macrosomia, shoulder dystocia, birth trauma, and operative deliveries [36].

Neonatal complications

In this study, the most common neonatal complication was LBW, which was reported in 36 patients, followed by hypoglycemia (9), jaundice (7). A similar type of neonatal complications was found among neonates born to GDM mothers; the prevalence of hypoglycemia was 4.5% in Prakash et al. [13] and 8% in Dudhwadkar et al. [27]. Stewart et al. [14] explain that hypoglycemia in neonates results from hyperinsulinism, which inhibits usual counter-regulatory responses such as gluconeogenesis, lipolysis, glycogenolysis, and fatty acid oxidation. Similar findings were reported by Karkia et al., who identified hypoglycemia and jaundice as neonatal complications in GDM patients [37]. In a study by Misra et al., it was shown that the most common complication among neonates born to GDM mothers was hyperbilirubinemia (16.67%) [38]. The basis for hypocalcemia in infants of diabetic mothers has been proposed as functional hypoparathyroidism [14].

Limitations of the study

The data was collected from a regional hospital in Mauritius. A multicentric study will represent the prevalence of GDM and its clinical outcome across Mauritius. Additionally, the absence of routine neonatal cardiac echocardiography may lead to lower detection of congenital heart anomalies. Neonatal outcomes are recorded only at birth or shortly after, so long-term complications or metabolic outcomes cannot be assessed. Due to the low prevalence of the disease, the study had a relatively small sample size. As a result, many findings, although clinically relevant, did not reach statistical significance.

What is already known on this topic

The global prevalence of GDM was 14.7% based on IADPSG (International Association of Diabetes and Pregnancy Study Groups) criteria. According to the International Diabetes Federation, Mauritius ranked as the twelfth highest disease-burdened country in the world. According to the noncommunicable disease survey report 2021, the prevalence of T2DM accounts for 19.9% in Mauritius, and it affects one in every five Mauritian people. Though data is available for DM in Mauritius, there is a lack of data and research on GDM in Mauritius. Considered as one of the most common complications of pregnancy, GDM has been studied to cause several maternal and neonatal adverse outcomes.

What this study adds

Gestational diabetes in Mauritius has been an unexplored field of study. There is a significant dearth of data on the prevalence, as well as fetal, neonatal, and maternal outcomes related to GDM in Mauritius. This study will bridge the knowledge gap to determine the prevalence of GDM in Mauritius, identify the risk factors predisposing Mauritian women to GDM, and assess maternal and fetal/neonatal complications of GDM. The findings of this study will help in the prevention of these complications by providing information to healthcare workers, the Ministry of Health and Wellness, and other nongovernmental organizations. 

## Conclusions

The prevalence of GDM was found to be 16% in Mauritius. A total of 52.2% patients belonged to the age group 20-30 years and were housewives (52.2%) by occupation. Most of them followed Christianity (58.6%). About 64.5% of the patients had a positive family history of DM, followed by other comorbidities (10.8%). GDM may cause significant maternal and neonatal complications. This study bridges the existing knowledge gap by determining the prevalence of GDM in Mauritius and identifying the risk factors specific to Mauritian women. The most common neonatal complication was LBW, followed by hypoglycemia, and jaundice. The most common maternal complications were preterm delivery, followed by recurrent UTI and gestational hypertension. Therefore, it is of utmost importance that all pregnant women are universally screened between 24 and 28 weeks in order to achieve good glycemic control during pregnancy.

## Appendices

**Table 6:** Questionnaire on the sociodemographic profile and clinical outcomes of gestational diabetes mellitus: a regional hospital-based cross-sectional study from Mauritius Credits: Sheena Bharati Auckloo

Patient number:	
Address:	
A. Sociodemographic details of patients with gestational diabetes mellitus	
a. Age	ii. <20 years
	ii. 20-30 years
	iii. >30 years
b. Educational level	i. Primary
	ii. Secondary
	iii. Tertiary
	iv. No education
c. Occupation	i. Housewife
	ii. Skilled worker
	iii. Unskilled worker
	iv. Professional
d. Family income	i. 0-9,000 MUR/month
	ii. 9,000-25,000 MUR/month
	iii. >25,000 MUR/month
e. Ethnicity	i. Christian
	ii. Hindu
	iii. Muslim
f. Marital status	i. Single
	ii. Married
	iii. Cohabitating
B. Risk factors of patients with gestational diabetes mellitus	
a. Smoking status	i. Smoker
	ii. Ex-smoker
	iii. Passive
	iv. Nonsmoker
b. Alcohol consumption	i. Yes
	ii. No
c. Parity	i. Multiparous
	ii. Uniparous
	iii. Nulliparous
d. Past GDM	i. Yes
	ii. No
e. Obesity	i. Normal weight
	ii. Obese class 1
	iii. Obese class 2
	iv. Obese class 3
	v. Overweight
	vi. Underweight
	i. Normal weight
C. Clinical outcomes of gestational diabetes mellitus	
a. Type of delivery	i. Normal vaginal delivery
	ii. Assisted delivery
	iii. Emergency cesarean section
	iv. Elective cesarean section
b. Pregnancy outcome	i. Alive
	ii. Intrauterine death (IUD)
	iii. Stillbirth
c. ICU admission	i. Yes
	ii. No
d. Treatment (Rx)	i. Diet
	ii. Metformin
	iii. Insulin
	iv. No treatment
e. Maternal complication	i. Yes
	ii. No
f. Type of maternal complications	i. Polyhydramnios
	ii. Preterm induced labour
	iii. Pregnancy-induced hypertension
	iv. Postpartum hemorrhage
	v. Premature rupture of membranes
	vi. Recurrent monilial infections
	vii. Recurrent urinary tract infection
	viii. Miscarriage
g. Neonatal complication	i. Yes
	ii. No
h. Type of neonatal complications	i. Small for gestational age
	ii. Large for gestational age
	iii. Jaundice
	iv. Hypoglycemia
	v. Shoulder dystocia
	vi. Congenital abnormality
	vii. Respiratory distress syndrome
	viii. Cardiac complication
	ix. Sepsis
i. Birth weight	i. Normal weight
	ii. Overweight
	iii. Underweight
